# Photoelectrochemical Selective Oxidation of Glycerol to Glyceraldehyde with Bi-Based Metal–Organic-Framework-Decorated WO_3_ Photoanode

**DOI:** 10.3390/nano13101690

**Published:** 2023-05-21

**Authors:** Yoonsung Jung, Seungkyu Kim, Hojoong Choi, Yunseul Kim, Jun Beom Hwang, Donghyeon Lee, Yejoon Kim, Jun-Cheol Park, Dong-Yu Kim, Sanghan Lee

**Affiliations:** 1 School of Materials Science and Engineering, Gwangju Institute of Science and Technology, Gwangju 61005, Republic of Korea; yoondo8557@gist.ac.kr (Y.J.); ggsg1014@gmail.com (S.K.); libertad@gm.gist.ac.kr (H.C.); yunseul.kim@chem.ox.ac.uk (Y.K.); pluto134340@gm.gist.ac.kr (J.B.H.); leedh@gm.gist.ac.kr (D.L.); yejoon0205@gm.gist.ac.kr (Y.K.); j-c.park@gist.ac.kr (J.-C.P.); kimdy@gist.ac.kr (D.-Y.K.); 2 Research Center for Innovative Energy and Carbon Optimized Synthesis for Chemicals (Inn-ECOSysChem), Gwangju Institute of Science and Technology, 123 Cheomdan-gwagiro, Buk-gu, Gwangju 61005, Republic of Korea

**Keywords:** photoelectrochemical, WO_3_, glycerol, oxidation, glyceraldehyde

## Abstract

The conversion of glycerol to high-value-added products via photoelectrochemical (PEC) oxidation has emerged as a promising approach for utilizing a sustainable and clean energy source with environmental and economic benefits. Moreover, the energy requirement for glycerol to produce hydrogen is lower than that for pure water splitting. In this study, we propose the use of WO_3_ nanostructures decorated with Bi-based metal–organic frameworks (Bi-MOFs) as the photoanode for glycerol oxidation with simultaneous hydrogen production. The WO_3_-based electrodes selectively converted glycerol to glyceraldehyde, a high-value-added product, with remarkable selectivity. The Bi-MOF-decorated WO_3_ nanorods enhanced the surface charge transfer and adsorption properties, thereby improving the photocurrent density and production rate (1.53 mA/cm^2^ and 257 mmol/m^2^·h at 0.8 V_RHE_). The photocurrent was maintained for 10 h, ensuring stable glycerol conversion. Furthermore, at 1.2 V_RHE_, the average production rate of glyceraldehyde reached 420 mmol/m^2^·h, with a selectivity of 93.6% between beneficial oxidized products over the photoelectrode. This study provides a practical approach for the conversion of glycerol to glyceraldehyde via the selective oxidation of WO_3_ nanostructures and demonstrates the potential of Bi-MOFs as a promising cocatalyst for PEC biomass valorization.

## 1. Introduction

The development of sustainable and environmentally friendly energy sources has become crucial in the effort to replace fossil fuels, particularly in the face of the increasing global demand for energy and the impacts of climate change [1,2,3,4]. A report by the United Nations Food and Agriculture Organization (OECD/FAO) shows that the annual production and consumption of biodiesel are increasing worldwide [5]. However, a significant amount of waste glycerol is generated as a by-product of biodiesel manufacturing [6,7,8]. Various technologies are being explored to maximize the benefits of biodiesel by converting low-cost glycerol into higher-value compounds. Most conversion methods, including reforming [9,10], hydrogenolysis [11,12], dehydration [13,14], esterification [15,16], etherification [17,18], oligomerization [19,20], carboxylation [21,22], and oxidation [23,24,25,26], have limitations such as the requirement for an additional energy source or the production of CO_2_ during the conversion process. However, photoelectrochemical (PEC) oxidation is a promising method for glycerol conversion, which is an environmentally friendly and straightforward process for directly transferring solar energy into the target material [27,28,29,30]. In addition, PEC oxidation of glycerol-containing systems is more aggressive than pure water splitting. Glycerol, which contains three hydroxyl groups, is kinetically easy to oxidize and thermodynamically requires less energy to oxidize than water [31,32,33]. Moreover, depending on the degree of glycerol oxidation, high-value-added products can be obtained from glycerol conversion. Glycerol oxidation produces mainly glyceraldehyde (GAD, C_3_H_6_O_3_), dihydroxyacetone (DHA, C_3_H_6_O_3_), glyceric acid, tartronic acid, glycolic acid, formic acid, and oxalic acid [34,35]. All products, except for formic acid, are more valuable than glycerol, which is commonly used in cosmetics and pharmaceuticals [36,37]. As an emerging high-value-added compound, GAD is used as a non-toxic cross-linking agent for anticancer and antibiotic pharmaceuticals and as a self-tanning agent material for skin care products in cosmetics [38,39]. Hence, the total energy required to simultaneously produce both high-value compounds and hydrogen can be reduced by replacing the oxidation of water with glycerol.

Recently, various metal oxides such as BiVO_4_ [40,41], TiO_2_ [42,43], ZnO [44,45], and WO_3_ [46,47] have been investigated to enhance the photocatalytic performance and to understand the glycerol conversion mechanism under the PEC reaction. Among these metal oxides, WO_3_ is an attractive photoanode for PEC glycerol oxidation due to its appropriate band gap (2.5–2.8 eV), hole diffusion length (150 nm), electron mobility (12 cm^2^/V·s), and valence band edge position (3.0 V vs. a reversible hydrogen electrode (RHE)) [48,49,50,51]. However, its major drawbacks, such as its rapid recombination of photoelectron–hole pairs and poor charge separation, hinder the improvement of the PEC performance [52]. To overcome these issues, morphology control [53,54], surface modification [55,56], heterojunction construction [57,58], doping [59,60], and oxygen evolution cocatalyst (OEC) loading [61,62] can be applied. Among these methods, loading OECs onto nanostructured WO_3_ photoanodes is effective in increasing the active area and promoting the charge transfer of glycerol [63,64]. As a crystalline material comprising metal and organic ligands, metal–organic frameworks (MOFs) have been considered promising OECs owing to their high specific surface areas, multiple active sites, tunable structures, good adsorptivity, and good catalytic stability [65,66,67,68]. However, most MOFs exhibit poor electrical conductivity and photoreactivity, and MOFs collapse when exposed to an aqueous environment [69,70,71]. Recently, some studies have reported that these problems can be solved by adjusting the metal cations of MOFs [72,73,74,75,76,77]. Bi-based MOFs (Bi-MOFs), which exhibit a stable and good charge transfer ability, in an aqueous environment are suitable for glycerol oxidation [77,78]. The oxidation process of cations can be influenced by the metal present in MOFs. In the case of Bi ions, the strong electrostatic attraction between the cation and the oxygen of the hydroxyl group in glycerol makes it easier to oxidize [40]. Moreover, the geometrically flexible coordination modes of Bi are advantageous for MOF configurations, and two regular helix chains permit the visible-light response of Bi-MOFs [78,79].

In this study, Bi-MOF-decorated WO_3_ (Bi-MOF/WO_3_) nanostructures were developed for the efficient production of high-value-added products from glycerol. A solvothermal process was employed for the synthesis of WO_3_ and Bi-MOFs to form a homogeneous nanostructure. The photoreactivities of the prepared samples were optimized by modulating the degree of surface decoration of the Bi-MOFs. Consequently, the photocurrent density of the Bi-MOF/WO_3_ photoanodes was improved to 0.46 mA/cm^2^ compared to that of the bare WO_3_ photoanodes (0.17 mA/cm^2^) at 0.8 V_RHE_ without glycerol. In addition, the photocurrent of the Bi-MOF/WO_3_ photoanodes was further improved to 1.53 mA/cm^2^ through the addition of glycerol, which is kinetically more easily oxidized than water. Notably, the Bi-MOFs loaded on the WO_3_ photoanodes improved the PEC performance in the oxidation of glycerol as compared to the bare WO_3_ photoanodes. The Bi-MOF/WO_3_ photoanodes (1.53 mA/cm^2^ at 0.8 V_RHE_) exhibited a higher photocurrent than the bare WO_3_ photoanodes (1.14 mA/cm^2^ at 0.8 V_RHE_), suggesting that Bi-MOFs facilitate not only conventional PEC water oxidation but also PEC glycerol oxidation. Only glycerol was involved in the oxidation of the Bi-MOF/WO_3_ photoanodes, and GAD and DHA were the main products. GAD was mainly obtained via PEC glycerol oxidation using Bi-MOF/WO_3_, with production rates of 257 mmol/m^2^·h (0.8 V_RHE_) and 420 mmol/m^2^·h (1.2 V_RHE_). In particular, the comparison of the faradaic efficiencies of the main products revealed that ~94% of the main products consisted of GAD. Therefore, PEC glycerol oxidation using Bi-MOF/WO_3_ photoanodes can produce not only the high-value-added compound GAD with high selectivity but also an environmentally friendly fuel such as H_2_. This research is expected to provide a strategy to improve the photoreactivity, selectivity, and stability of WO_3_ photoanodes by using MOF cocatalysts for PEC glycerol oxidation.

## 2. Materials and Methods

### 2.1. Materials

Hydrochloric acid (HCl, 37%, Sigma-Aldrich, Vienna, Austria), ammonium paratungstate ((NH_4_)_10_H_2_(W_2_O_7_)_6_, 99.99%, Sigma-Aldrich, Tokyo, Japan), hydrogen peroxide (H_2_O_2_, 30%, Sigma-Aldrich, Madrid, Spain), bismuth(III) nitrate pentahydrate (Bi(NO_3_)_3_·5H_2_O, 98%, Sigma-Aldrich, Mexico City, Mexico), methanol (MeOH, 99.8%, Sigma-Aldrich, St. Louis, MO, USA), N,N-dimethylformamide (DMF, 99.8%, Sigma-Aldric, Ankara, Turkey), trimesic acid (C_6_H_3_(CO_2_H)_3_, 95%, Sigma-Aldrich, Shanghai, China), sodium sulfate (Na_2_SO_4_, 99.0%, Sigma-Aldrich, Bangalore, India), glycerol (C_3_H_8_O_3_, 99+%, Alfa Aesar, Selangor, Malaysia) and sulfuric acid (Alfa Aesar, Haverhill, MA, USA). All materials were analytical grade and used without further purification.

### 2.2. Fabrication of WO_3_ and Bi-MOFs

**Figure 1:**
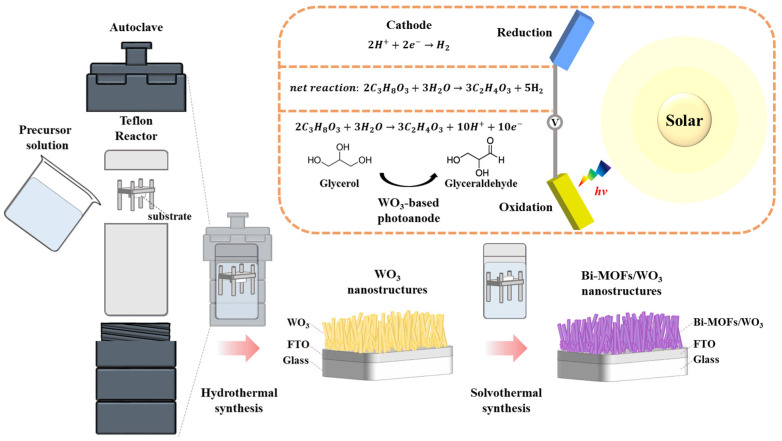
Schematic of the WO_3_ and Bi-MOF nanostructures and redox reaction.

Figure 1 shows the synthesis of the WO_3_ and Bi-MOF nanostructures. WO_3_-based electrodes exhibit remarkable selectivity in the conversion of glycerol to GAD, which is a high-value-added product. The precursor solution used for the synthesis of the WO_3_ nanostructure was prepared by adding 0.687 mL of HCl to 69 mL of deionized (DI) water, containing 0.6048 g (0.20 mmol) of dissolved ammonium paratungstate. The precursor solution was left at room temperature for more than 4 h. Subsequently, 1.452 mL of H_2_O_2_ was added to the precursor solution to dissolve the precipitated tungsten acid. After the precursor solution was prepared, fluorine-doped tin oxide (FTO) glass was transferred to a Teflon-lined stainless-steel autoclave with the conductive surface facing down in the precursor solution. Then, hydrothermal synthesis was performed at 180 °C for 4 h. After the reaction, the Teflon-lined stainless-steel autoclave was cooled naturally at room temperature. The as-obtained WO_3_ was carefully cleaned with DI water and annealed at 500 °C for 2 h in air.

A series of Bi-MOF catalysts were synthesized using the solvothermal method. The precursor solution used for the synthesis of the Bi-MOFs was prepared by dissolving 0.283 g (0.58 mmol) of bismuth(III) nitrate pentahydrate and 0.230 g (1.09 mmol) of trimesic acid in a solvent containing 45 mL of MeOH and 15 mL of DMF. Then, the as-prepared WO_3_ samples were immersed in the prepared solution for 10, 30, and 60 min at room temperature. The as-obtained Bi-MOF/WO_3_ was washed with methanol and dried in air at 60 °C. Finally, the as-prepared Bi-MOF/WO_3_ was annealed at 200 °C for 1 h.

### 2.3. Material Characterization

The structural characteristics of the Bi-MOF/WO_3_ photoelectrode were identified via X-ray diffraction (XRD, Bruker AXS, Karlsruhe, Germany) using Cu Kα radiation (λ = 1.5406 Å) at 40 kV and 40 mA. To confirm the nanostructure morphology, field-emission scanning electron microscopy (FESEM, Hitachi S-4700, Hitachi, Tokyo) images were recorded. The elemental composition of Bi-MOF/WO_3_ was investigated by recording energy-dispersive spectroscopy (EDS) mapping images on a high-angle annular dark-field scanning transmission electron microscope (HAADF-STEM, Tecnai G2 F30 S-twin, FEI) at 300 kV and an X-ray photoelectron spectroscopy (XPS, NEXSA, Thermo Fisher Scientific, Waltham, MA, USA) system. Ultraviolet–visible (UV–Vis) absorption spectra of the photoanodes were measured using a Shimadzu UV–Vis spectrophotometer (UV-2600, Tokyo, Japan).

### 2.4. PEC Measurements

Electrochemical and photoelectrochemical characteristics were investigated using a potentiostat (Ivium-n-Stat, Ivium Technologies, Eindhoven, The Netherlands) with a 150 W Xe lamp (Model 10500, ABET Technology, Milford, CT, USA). The light intensity was calibrated using a photodiode (Bunkokeiki Co., Ltd. Tokyo, Japan) under 1-sun intensity (100 mW/cm^2^). All PEC measurements were performed in a three-electrode system inside a quartz cell, where a Pt coil, Ag/AgCl electrode, and photoanodes were used as the counter electrode (CE), reference electrode (RE), and working electrode (WE), respectively.

The potentials vs. the Ag/AgCl electrode were converted to those vs. RHE using the Nernst equation:

$$E_{RHE}=E_{Ag/AgCl}+0.059\times \mathrm{pH}+0.197$$

where *E*_RHE_ is the converted potential, and *E*_Ag/AgCl_ is the reference potential of the Ag/AgCl electrode. To use photoanodes as the WE, a Cu wire and FTO were connected using Ag paste. After the Ag paste was dried, FTO and the Cu wire were sealed with epoxy resin to separate the electrodes from the electrolyte.

The pH of all 0.5 M Na_2_SO_4_ electrolytes was calibrated to 2 using sulfuric acid, regardless of the addition of glycerol. Linear sweep voltammetry (LSV) was performed at a scan rate of 20 mV/s and a scan step size of 10 mV. The corresponding Tafel slopes were calculated using the Tafel equation, as follows:

η=b×log(J)+a

where η is the overpotential, b is the Tafel slope, and J is the current density.

Electrochemical impedance spectroscopy (EIS) was performed at 0.8 V_RHE_ in a frequency range of 100 kHz to 0.5 Hz under illumination.

A monochromator (Mmac 200, Dongwoo OPTRON, Gwangju, Korea) was used at wavelengths ranging from 300 to 550 nm for measurements of the incident photon-to-current efficiency (IPCE), which was calculated using the following equation:

$$\mathrm{IPCE}=\frac{1240\times J_{ph}}{\lambda \times P}\times 100\%$$

where *J*_ph_ is the photocurrent density, λ is the wavelength, and P is the incident light intensity.

Faradaic efficiency (η) was calculated by measuring the amount of product estimated via high-performance liquid chromatography (HPLC, Waters Alliance 2695, Aminex-HPX-87H column, Hercules, CA, USA) and gas chromatography (GC, 6500GC, 5 Å molecular sieve column).

High-purity Ar gas was injected into a sealed quartz cell at a rate of 20 mL/min, followed by GC analysis to determine the gas generated using a thermal conductivity detector (TCD). The temperatures of the oven and detector were maintained at 50 °C and 150 °C, respectively. The faradaic efficiency of the produced gas was calculated using the following equation:

$$\mathrm{Faradaic}\;\mathrm{efficiency}\;\left(\eta \right)=\frac{zFP/RT\times \frac{v}{60}\times \frac{n}{1,000,000}}{i}$$

where *z* is the number of charges that produces a molecule of the product, *F* is the Faraday constant, *P* is the pressure, *R* is the gas constant, *T* is the temperature, *v* is the volume flow rate, *n* is the concentration of gas detected via GC, and *i* is the current. The *z* values of hydrogen and oxygen are 2 and 4, respectively, according to the following reaction formula:

$$2H_{2}O\to 4H^{+}+4e^{-}+O_{2},\;2H^{+}+2e^{-}\to H_{2}$$

Twenty microliters of the electrolyte in which the electrochemical reaction occurred was subjected to HPLC analysis by flowing 5 mM sulfuric acid into the UV detector (Waters 2487 Dual λ Absorbance Detector) at 0.5 mL/min with an Aminex HPX 87-H column (300 mm × 7.8 mm). The oven temperature was maintained at 60 °C, and the UV detector wavelength was maintained constant at 210 nm to detect the products obtained via oxidation. The faradaic efficiency of the products in the liquid was calculated using the following equation:

$$\mathrm{Faradaic}\;\mathrm{efficiency}=\frac{z\times mol\times \frac{V}{u}}{Q}$$

where *mol* is the number of moles of products detected via liquid chromatography (LC), *V* is the total volume of the electrolyte, *u* is the injection volume of LC, and Q is the total charge passed through the electrode. The *z* value of GAD and DHA is 2, according to the following reaction formula:

$$\mathrm{Glyceraldehyde}\;\&\;\mathrm{Dihydroxyacetone}\;(\mathrm{GAD}/\mathrm{DHA}):\;C_{3}H_{8}O_{3}\to C_{3}H_{6}O_{3}+2H^{+}+2e^{-}.$$

## 3. Results and Discussion

**Figure 2:**
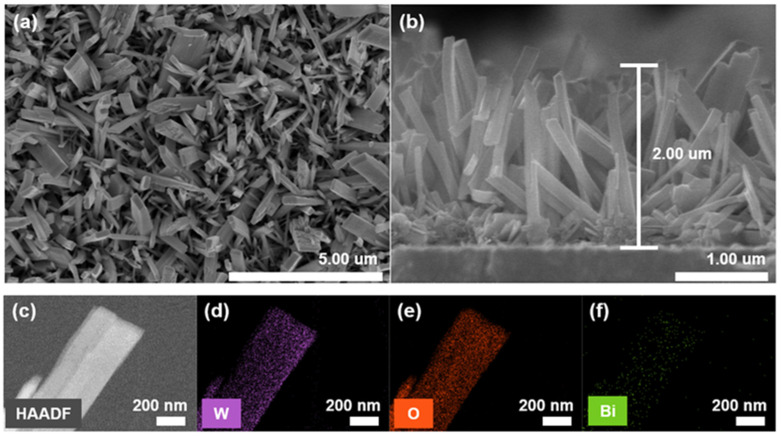
Structural characteristics of Bi-MOF/WO_3_ on FTO. (**a**) Top-view SEM images. (**b**) Cross-sectional SEM images. (**c**) HAADF-STEM image, and (**d**–**f**) corresponding structures and elemental distributions.

The morphologies of the WO_3_ and Bi-MOF/WO_3_ nanostructures were investigated via FESEM characterization. Figure 2a,b show the top-view and cross-sectional images of the Bi-MOF/WO_3_ nanostructures, respectively. A WO_3_ nanostructure with a length of 1–2 μm was formed on the FTO substrate. Vertically aligned WO_3_ nanostructures were successfully synthesized on the FTO substrate without any seed layer. The FESEM images of the Bi-MOF/WO_3_ (Figure 2a) and WO_3_ nanostructures (Figure S1) were the same, indicating that the as-deposited WO_3_ was stable during the synthesis of the Bi-MOFs. In addition, HAADF-STEM and STEM-EDS mapping images were recorded to verify the microscopic structures and elemental distributions. The dispersion of Bi elements on the WO_3_ surface in the mapping images confirmed the successful synthesis of the Bi-MOFs (Figure 2c–f).

**Figure 3:**
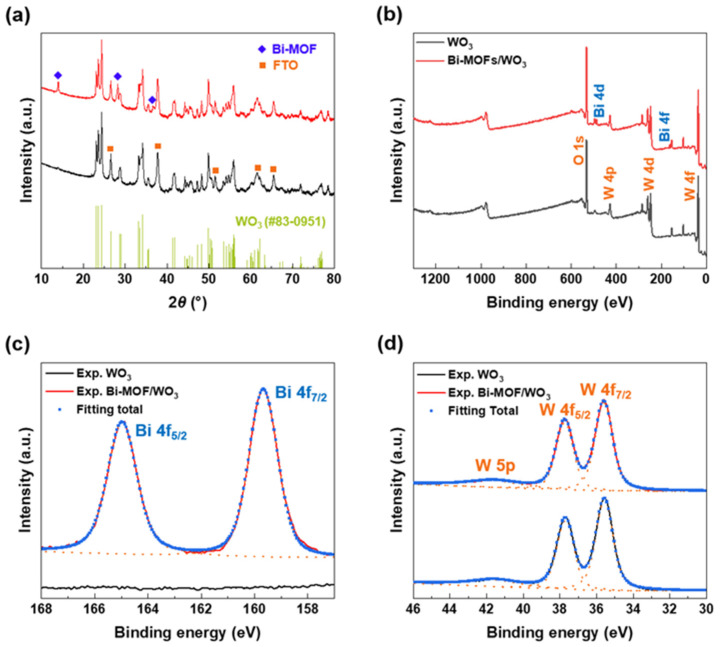
(**a**) XRD *θ*−2*θ* scans. (**b**) XPS survey spectra. (**c**) Bi 4f XPS core-level spectra. (**d**) W 4f XPS core-level spectra of WO_3_ and Bi-MOF/WO_3_ photoanodes.

To confirm the crystalline phases of the Bi-MOF/WO_3_ crystal structure, XRD analysis was conducted. The crystalline phases of the Bi-MOFs (denoted by ◆) were observed at 2*θ* values of 13.99°, 28.22°, and 36.61°, as shown in the XRD patterns in Figure 3a [78]. When the synthesis time of the Bi-MOFs was 30 min, the highest-intensity peak of the Bi-MOFs was observed due to the improved crystallinity (Figure S2). In contrast, when the synthesis time was 60 min, the crystalline phase of the Bi-MOFs was not formed. At 2 *θ* values of 37.8°, 51.55°, 65.5°, and 78.6°, peaks were exhibited by the FTO substrate (denoted by ■). The peaks observed for WO_3_ could be accurately indexed to the monoclinic phase [80]. The highest-intensity peak at 24.3° corresponded to the (200) plane. The strength and position of the peaks corresponding to the monoclinic phase were consistent in the synthesis of the Bi-MOFs.

The surface composition of Bi-MOF/WO_3_ and WO_3_ was identified through XPS narrow-scan analysis of W 4f, W 5p, and Bi 4f (Figure 3b). The orange dashed line represents the fitting results for each split orbital state, while the blue points indicate the points obtained when the orbital states have converged. Doublet peaks were observed at W 4f_5/2_ (37.8 eV) and W 4f_7/2_ (35.6 eV) for WO_3_ and Bi-MOF/WO_3_ (Figure 3d) [81]. The Bi-MOFs did not affect the configuration of WO_3_ (Figure 3d), which was confirmed by the constant position and similar shapes of the peaks. By contrast, the peaks of Bi 4f_5/2_ (165.0 eV) and Bi 4f_7/2_ (159.7 eV) were only observed for Bi-MOF/WO_3_ and not for the bare WO_3_ (Figure 3c) [82]. These results confirm that Bi-MOFs can be successfully synthesized on bare WO_3_.

To investigate the light adsorption capability of the photoelectrodes, the UV–Vis light absorbance spectra and Tauc plots are depicted in Figure S3. There were no significant differences in the UV–Vis absorbance spectra between the WO_3_ and Bi-MOF/WO_3_ photoelectrodes. The absorption spectra were similar between the two electrodes, indicating that the presence of the Bi-MOF catalyst did not contribute to the decrease in the light adsorption efficiency. Furthermore, based on the Tauc plots, it was determined that the band gap of the photoelectrode was about 2.7 eV. This indicates that the photoelectrode is suitable as a candidate for PEC glycerol oxidation.

The PEC performance was evaluated on the three-electrode system under AM 1.5 G illumination (100 mW/cm^2^) with a Pt coil and Ag/AgCl (sat. KCl) as the CE and RE, respectively. Figure S4 shows the LSV polarization curve vs. the synthesis time of the Bi-MOFs. The WO_3_ photoanode with Bi-MOFs synthesized for 30 min exhibited the highest photocurrent density of 1.01 mA/cm^2^ at 0.8 V_RHE_ and 1.81 mA/cm^2^ at 1.2 V_RHE_. In addition, the relationship between the crystallinity of the Bi-MOFs and the charge transfer resistance was confirmed by the EIS curves, followed by the fitting of the Nyquist plots of the photoanode to an equivalent circuit. The charge transfer resistance of the WO_3_ photoanode with Bi-MOFs synthesized for 30 min was lower than that of the Bi-MOFs synthesized for 10 and 60 min and the bare WO_3_. Hence, the WO_3_ photoanode with Bi-MOFs synthesized for 30 min was selected as the optimal photoanode, and its higher PEC performance was attributed to it having the most efficient cocatalyst with the highest crystallinity, i.e., the Bi-MOFs synthesized for 30 min, as confirmed by the XRD data (Figure S3).

**Figure 4:**
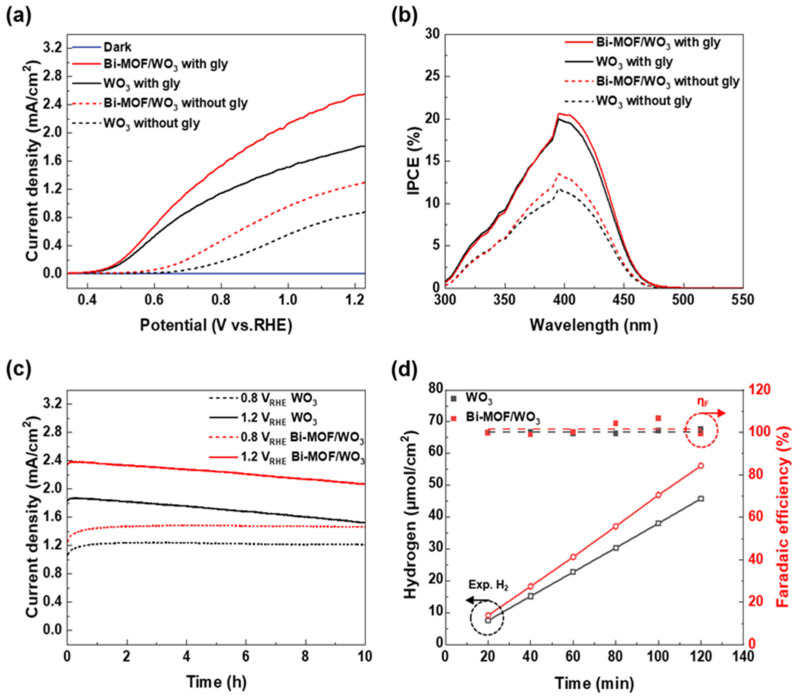
PEC performance of WO_3_ and Bi-MOF/WO_3_ in 0.5 M Na_2_SO_4_ electrolytes, with the pH adjusted to 2 using sulfuric acid, with and without 2 M glycerol. (**a**) Representative LSV curves. (**b**) IPCE at 0.8 V_RHE_. (**c**) Chronoamperometric measurement used to evaluate PEC stability with 2 M glycerol, and (**d**) the corresponding faradaic efficiency and production rate of hydrogen at 0.8 V_RHE_.

Figure 4a shows representative LSV curves of the photoanodes with and without glycerol. In the electrolyte without glycerol, the photocurrent density of the bare WO_3_ was 0.85 mA/cm^2^ (at 1.2 V_RHE_), while that of Bi-MOF/WO_3_ increased to 1.26 mA/cm^2^ (1.2 V_RHE_). Furthermore, glycerol with three hydroxyl groups was more easily oxidized than water, resulting in a further improvement of the PEC performance of the glycerol-containing electrolytes [31,83]. Through the addition of 2 M glycerol in the electrolytes, the photocurrent density of the bare WO_3_ was 1.78 mA/cm^2^ (1.2 V_RHE_), and the photocurrent density of Bi-MOF/WO_3_ was 2.53 mA/cm^2^ (1.2 V_RHE_); this result reveals a twofold performance improvement in comparison with that of the electrolyte without glycerol. By contrast, the onset potential of the electrolyte with glycerol was shifted to more negative potentials than those of the electrolyte without glycerol. The effect of photocurrent enhancement is more pronounced at lower bias potentials, such as 0.8 V_RHE_. The photocurrent density of the bare WO_3_ was improved from 0.17 mA/cm^2^ to 1.14 mA/cm^2^ at 0.8 V_RHE_, and the photocurrent density of Bi-MOF/WO_3_ was improved from 0.46 mA/cm^2^ to 1.53 mA/cm^2^ at 0.8 V_RHE_. By incorporating glycerol and Bi-MOFs into WO_3_, the photocurrent of WO_3_ was improved by a factor of 3 at 1.2 V_RHE_ and by factor of 8 at 0.8 V_RHE_. These results suggest that the Bi-MOFs and glycerol significantly contributed to the PEC performance improvement of the WO_3_ photoanodes. Figure S5 shows the corresponding Tafel plots of the photoanodes. The Tafel slope of the photoanodes with glycerol (116, 121 mV/dec) was smaller than that of the photoanodes without glycerol (182, 194 mV/dec). Electrolytes containing glycerol exhibit lower Tafel slopes due to the fast charge transfer kinetics of alcohols such as glycerol [84,85]. The Bi-MOF/WO_3_ photoanode in the electrolyte with glycerol exhibited the smallest Tafel slope (116 mV/dec). It was confirmed that the oxidation by the photoanodes was facilitated by the addition of glycerol and the loading of the Bi-MOFs, which drastically improved the PEC performance of the WO_3_ photoanodes.

To evaluate the dependence of the PEC performance on the wavelength, the IPCE of the WO_3_ photoanodes was measured at wavelengths in the range of 300–550 nm. When the PEC performance was measured in an electrolyte without glycerol at 0.8 V_RHE_, the IPCE of the bare WO_3_ was 11.8% at 395 nm, while that of Bi-MOF/WO_3_ was improved to 13.5% (Figure 4b). The addition of glycerol in an electrolyte also improved the IPCE, similar to the LSV results (Figure 4a). At a wavelength of 395 nm, WO_3_ exhibited an IPCE of 19.99%, whereas Bi-MOF/WO_3_ exhibited an improved IPCE of 20.63%. The IPCE measured at 1.2 V_RHE_ was greater than that measured at 0.8 V_RHE_ (Figure S6). Without glycerol, the IPCE values for WO_3_ and Bi-MOF/WO_3_ were 22.7% and 23.9%, respectively. In the presence of glycerol, the IPCE values for WO_3_ and Bi-MOF/WO_3_ increased to 33.4% and 33.8%, respectively.

The PEC stability of the photoanodes was evaluated for 10 h with an electrolyte containing 2 M glycerol (Figure 4c). The photocurrent density of the photoanodes increased from the initial value and remained constant at 0.8 V_RHE_ for 10 h. At 1.2 V_RHE_, the photocurrent densities of WO_3_ and Bi-MOF/WO_3_ were maintained at 82% and 87% of their initial photocurrent after 10 h, respectively. To analyze the PEC performance and selectivity of the photoanodes in terms of gas production, the amount of H_2_ and O_2_ generated from the photoanodes was determined, and their faradaic efficiencies were calculated. For all electrodes, the faradaic efficiency of H_2_ was ~100%, as glycerol did not interfere with the reduction to hydrogen (Figure 4d). At 0.8 V_RHE_, the average production rates of H_2_ for WO_3_ and Bi-MOF/WO_3_ were determined to be 22.9 and 28.1 μmol/cm^2^·h, respectively (Figure 4d). By increasing the applied potential to 1.2 V_RHE_, the average production rates for WO_3_ and Bi-MOF/WO_3_ were improved to 33.2 and 43.3 μmol/cm^2^·h, respectively, similar to the improved photocurrent density revealed in the chronoamperometric measurement (Figure S7). This was because the photogenerated holes from Bi-MOF/WO_3_ were used for glycerol oxidation rather than water oxidation. As shown in Figure S8, O_2_ gas was not detected, so the photocurrent in the oxidation reaction was 100% due to the photoelectrochemical reaction of glycerol.

**Figure 5:**
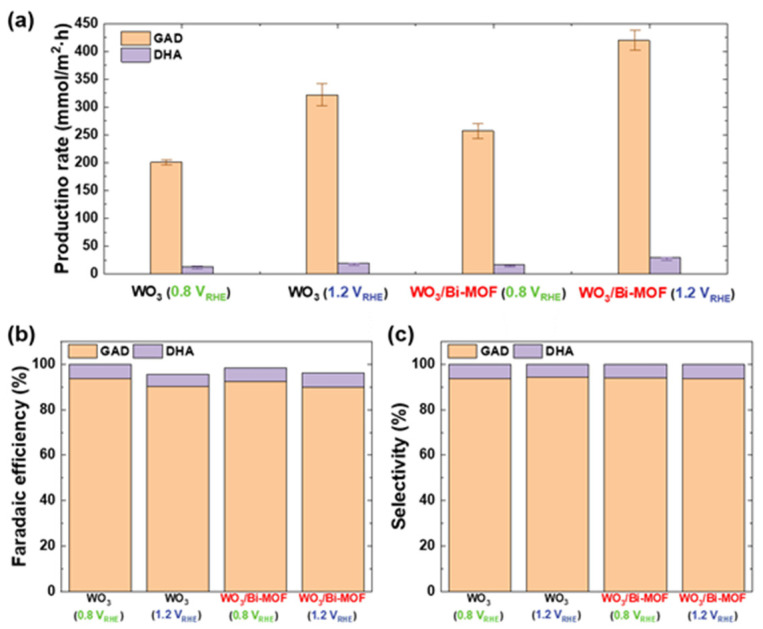
Oxidant products from WO_3_ and Bi-MOF/WO_3_: (**a**) production rate, (**b**) faradaic efficiency, and (**c**) selectivity at 0.8 V_RHE_ and 1.2 V_RHE_.

The product mixture generated by the PEC reaction for 10 h in an electrolyte with 2 M glycerol was analyzed via HPLC. Figure 5 and Table S1 show the quantitative analysis and faradaic efficiency of the products generated over WO_3_ and Bi-MOF/WO_3_, respectively. Glycerol oxidation products were identified via their retention times and mass spectroscopy, and quantitative analysis was performed through the calibration of each product using standard materials (Figure S9). GAD and DHA were detected as the main products without any other products. When glycerol was oxidized over WO_3_, GAD was produced in the highest amount, with 201 mmol/m^2^·h at 0.8 V_RHE_ and 322 mmol/m^2^·h at 1.2 V_RHE_. The improved PEC performance of Bi-MOF/WO_3_ was attributed to the more aggressive oxidation of glycerol over Bi-MOF/WO_3_ compared to that over WO_3_. The production of GAD increased to 257 mmol/m^2^·h (at 0.8 V_RHE_) and 420 mmol/m^2^·h (at 1.2 V_RHE_). Similarly, DHA production was increased from 13 mmol/m^2^·h to 16 mmol/m^2^·h at 0.8 V_RHE_, and from 19 mmol/m^2^·h to 29 mmol/m^2^·h at 1.2 V_RHE_, by the Bi-MOF catalyst. The total faradaic efficiency was maintained at ~100% at 0.8 V_RHE_, but decreased to 96.2% at 1.2 V_RHE_, despite the increase in the total production rate (Figure 5b). On the other hand, the selectivity of GAD was maintained regardless of the potential (94%) in the overall conversion efficiency (Figure 5c). As the PEC reaction continues and the concentration of GAD and DHA in the electrolyte increases, glycerol is over-oxidized and converted to CO_2_, a stable phase lower than the thermodynamic driving force of the two products [86]. However, Bi-MOF/WO_3_ maintained the selectivity of GAD in favor of converting glycerol to GAD, even in the presence of aggressive PEC reactions. To the best of our knowledge, our study reports the highest selectivity among catalysts for the conversion of glycerol to GAD, and this selective oxidation for PEC reactions has not been reported thus far. Table S2 shows the faradaic efficiency and selectivity with the detailed catalyst structures. As a result, by controlling the reactivity of glycerol, WO_3_ decorated with Bi-MOF cocatalysts could selectively produce GAD.

## 4. Conclusions

In conclusion, the utilization of Bi-MOF/WO_3_ nanostructures is a promising strategy for converting glycerol into high-value-added products and generating sustainable energy sources. The WO_3_-based electrodes exhibited high reactivity with glycerol during PEC glycerol oxidation, requiring less applied potential energy than water splitting. The addition of a Bi-MOF cocatalyst further improved the PEC performance by preventing electron–hole recombination and enhancing glycerol adsorption, resulting in a Bi-MOF/WO_3_ photocurrent density of 1.53 mA/cm^2^ at 0.8 V_RHE_. The PEC reaction was stable for 10 h, and the primary products of glycerol oxidation were GAD and DHA. One of the high-value-added products, GAD, was produced at a rate of 257 mmol/m^2^·h, with a selectivity of 4.7%. Increasing the applied potential to 1.2 V_RHE_ accelerated the oxidation reaction, leading to an improved photocurrent density and GAD production rate of 2.53 mA/cm^2^ and 420 mmol/m^2^·h, respectively. The selectivity of GAD remained high at 93.0%, even during the aggressive glycerol oxidation. In this regard, the use of Bi-MOF cocatalysts on WO_3_-based nanostructures is an effective strategy for the selective and efficient conversion of biomass into certain high-value-added products with enhanced PEC performance. These results have significant implications for the development of technologies for energy conversion systems and utilization.

## Data Availability

The data supporting the research in this study are available in the article.

## Supplementary Materials

The following supporting information can be downloaded at: https://www.mdpi.com/article/10.3390/nano13101690/s1, Figure S1: Top-view SEM images of WO_3_ on FTO; Figure S2: Out-of-plane *θ*−2*θ* scans of Bi-MOF/WO_3_ at different synthesis times of MOF; Figure S3: (a) UV-vis absorbance spectra, (b) Tauc plot; Figure S4: PEC performance of WO_3_ and Bi-MOF/WO_3_ in 0.5 M Na_2_SO_4_ electrolytes, with pH adjusted to 2 using sulfuric acid, with 0.1 M glycerol. (a) LSV polarization curves, (b) Nyquist plots with different synthesis times of MOF; Figure S5: Tafel plots of WO_3_ and Bi-MOF/WO_3_ in 0.5 M Na_2_SO_4_ electrolytes, with pH adjusted to 2 using sulfuric acid, with and without 2 M glycerol; Figure S6: IPCE values at 1.2 V vs. RHE with 2 M glycerol; Figure S7: Faradaic efficiency and production rate of hydrogen at 1.2 V vs. RHE; Figure S8: Faradaic efficiency and production rate of oxygen; Figure S9: (a) Liquid chromatography of produced glyceraldehyde, (b) calibration curve of standard glyceraldehyde (c) NMR spectra of produced dihydroxyacetone, (d) calibration curve of standard dihydroxy acetone. Table S1: Reaction rate: production rate and faradaic efficiency from WO3 and Bi-MOF/WO3; Table S2: Photocatalyst, and photoelectrode for the selective oxidation of glycerol [40,41,87,88,89,90,91].

## References

[1] Zou X., Zhang Y. (2015). Noble metal-free hydrogen evolution catalysts for water splitting. Chem. Soc. Rev..

[2] Roger I., Shipman M.A., Symes M.D. (2017). Earth-abundant catalysts for electrochemical and photoelectrochemical water splitting. Nat. Rev. Chem..

[3] Voloshin R.A., Rodionova M.V., Zharmukhamedov S.K., Veziroglu T.N., Allakhverdiev S.I. (2016). Review: Biofuel production from plant and algal biomass. Int. J. Hydrogen Energy.

[4] Singh D., Sharma D., Soni S., Sharma S., Sharma P.K., Jhalani A. (2020). A review on feedstocks, production processes, and yield for different generations of biodiesel. Fuel.

[5] OECD/FAO (2020). OECD-FAO Agricultural Outlook 2020–2029.

[6] Zhou C.C., Beltramini J.N., Fan Y.X., Lu G.M. (2008). Chemoselective catalytic conversion of glycerol as a biorenewable source to valuable commodity chemicals. Chem. Soc. Rev..

[7] Sheldon R.A. (2014). Green and sustainable manufacture of chemicals from biomass: State of the art. Green Chem..

[8] Yazdani S.S., Gonzalez R. (2007). Anaerobic fermentation of glycerol: A path to economic viability for the biofuels industry. Curr. Opin. Biotechnol..

[9] Zhang B., Tang X., Li Y., Xu Y., Shen W. (2007). Hydrogen production from steam reforming of ethanol and glycerol over ceria-supported metal catalysts. Int. J. Hydrogen Energy.

[10] Kondarides D.I., Daskalakim V.M., Patsoura A., Verykios X.E. (2007). Hydrogen Production by Photo-Induced Reforming of Biomass Components and Derivatives at Ambient Conditions. Catal. Lett..

[11] Maris E., Davis R. (2007). Hydrogenolysis of glycerol over carbon-supported Ru and Pt catalysts. J. Catal..

[12] Chaminand J., Djakovitch L., Gallezot P., Marion P., Pinel C. (2004). Glycerol hydrogenolysis on heterogeneous catalysts. Green Chem..

[13] Katryniok B., Paul S., Bellière-Baca V., Rey P., Dumeignil F. (2010). Glycerol dehydration to acrolein in the context of new uses of glycerol. Green Chem..

[14] Yang J., Huang L., Yi T., Wang X., Gao L., Liu W. (2022). Glycerin to Acrolein: Can Renewable Processes Challenge Traditional Processes?. Chem. Eng. Technol..

[15] Ochoa-Gómez J.R., Gómez-Jiménez-Aberasturi O., Maestro-Madurga B., Pesquera-Rodríguez A., Ramírez-López C., Lorenzo-Ibarreta L., Torrecilla-Soria J., Villarán-Velasco M.C. (2009). Synthesis of glycerol carbonate from glycerol and dimethyl carbonate by transesterification: Catalyst screening and reaction optimization. Appl. Catal. A Gen..

[16] Bancquart S., Vanhove C., Pouilloux Y., Barrault J. (2001). Glycerol transesterification with methyl stearate over solid basic catalysts. Appl. Catal. A Gen..

[17] Klepáčová K., Mravec D., Kaszonyi A., Bajus M. (2007). Etherification of glycerol and ethylene glycol by isobutylene. Appl. Catal. A Gen..

[18] Melero J.A., Vicente G., Paniagua M., Morales G., Muñoz P. (2012). Etherification of biodiesel-derived glycerol with ethanol for fuel formulation over sulfonic modified catalysts. Bioresour. Technol..

[19] Martin A., Richter M. (2010). Oligomerization of glycerol—A critical review. Eur. J. Lipid Sci. Technol..

[20] Barrault J., Clacens J.-M., Pouilloux Y. (2004). Selective Oligomerization of Glycerol over Mesoporous Catalysts. Top. Catal..

[21] Aresta M., Dibenedetto A., Nocito F., Pastore C. (2006). A study on the carboxylation of glycerol to glycerol carbonate with carbon dioxide: The role of the catalyst, solvent and reaction conditions. J. Mol. Catal. A Chem..

[22] Ezhova N.N., Korosteleva I.G., Kolesnichenko N.V., Kuz’min A.E., Khadzhiev S.N., Vasil’eva M.A., Voronina Z.D. (2012). Glycerol carboxylation to glycerol carbonate in the presence of rhodium complexes with phosphine ligands. Pet. Chem..

[23] Carrettin S., McMorn P., Johnston P., Griffin K., Hutchings G.J. (2002). Selective oxidation of glycerol to glyceric acid using a gold catalyst in aqueous sodium hydroxide. Chem. Commun..

[24] Carrettin S., McMorn P., Johnston P., Griffin K., Kiely C.J., Hutchings G.J. (2003). Oxidation of glycerol using supported Pt, Pd and Au catalysts. Phys. Chem. Chem. Phys..

[25] Mou H., Chang Q., Xie Z., Hwang S., Kattel S., Chen J.G. (2022). Enhancing glycerol electrooxidation from synergistic interactions of platinum and transition metal carbides. Appl. Catal. B Environ..

[26] Huang X., Guo Y., Zou Y., Jiang J. (2022). Electrochemical oxidation of glycerol to hydroxypyruvic acid on cobalt (oxy)hydroxide by high-valent cobalt redox centers. Appl. Catal. B Environ..

[27] Chen Z., Zhang G., Cao S., Chen G., Li C., Izquierdo R., Sun S. (2023). Advanced semiconductor catalyst designs for the photocatalytic reduction of CO_2_. Mater. Rep. Energy.

[28] He J., Liu P., Ran R., Wang W., Zhou W., Shao Z. (2022). Single-atom catalysts for high-efficiency photocatalytic and photoelectrochemical water splitting: Distinctive roles, unique fabrication methods and specific design strategies. J. Mater. Chem. A.

[29] Han X., Liu P., Ran R., Wang W., Zhou W., Shao Z. (2022). Non-metal fluorine doping in Ruddlesden–Popper perovskite oxide enables high-efficiency photocatalytic water splitting for hydrogen production. Mater. Today Energy.

[30] Xiao H., Liu P., Wang W., Ran R., Zhou W., Shao Z. (2022). Enhancing the photocatalytic activity of Ruddlesden-Popper Sr_2_TiO_4_ for hydrogen evolution through synergistic silver doping and moderate reducing pretreatment. Mater. Today Energy.

[31] Lu X., Xie S., Yang H., Tong Y., Ji H. (2014). Photoelectrochemical hydrogen production from biomass derivatives and water. Chem. Soc. Rev..

[32] Ibadurrohman M., Hellgardt K. (2014). Photoelectrochemical performance of graphene-modified TiO_2_ photoanodes in the presence of glycerol as a hole scavenger. Int. J. Hydrogen Energy.

[33] Mohapatra S.K., Raja K.S., Mahajan V.K., Misra M. (2008). Efficient Photoelectrolysis of Water using TiO_2_ Nanotube Arrays by Minimizing Recombination Losses with Organic Additives. J. Phys. Chem. C.

[34] Yang L., Li X., Chen P., Hou Z. (2019). Selective oxidation of glycerol in a base-free aqueous solution: A short review. Chin. J. Catal..

[35] Katryniok B., Kimura H., Skrzyńska E., Girardon J.-S., Fongarland P., Capron M., Ducoulombier R., Mimura N., Paul S., Dumeignil F. (2011). Selective catalytic oxidation of glycerol: Perspectives for high value chemicals. Green Chem..

[36] Skrzyńska E., Wondołowska-Grabowska A., Capron M., Dumeignil F. (2014). Crude glycerol as a raw material for the liquid phase oxidation reaction. Appl. Catal. A Gen..

[37] Dodekatos G., Schünemann S., Tüysüz H. (2018). Recent Advances in Thermo-, Photo-, and Electrocatalytic Glycerol Oxidation. ACS Catal..

[38] Jung K., Seifert M., Herrling T., Fuchs J. (2008). UV-generated free radicals (FR) in skin: Their prevention by sunscreens and their induction by self-tanning agents. Spectrochim. Acta Part A-Mol. Biomol. Spectrosc..

[39] Vandelli M.A., Rivasi F., Guerra P., Forni F., Arletti R. (2001). Gelatin microspheres crosslinked with D,L-glyceraldehyde as a potential drug delivery system: Preparation, characterisation, in vitro and in vivo studies. Int. J. Pharm..

[40] Liu D., Liu J.-C., Cai W., Ma J., Bin Yang H., Xiao H., Li J., Xiong Y., Huang Y., Liu B. (2019). Selective photoelectrochemical oxidation of glycerol to high value-added dihydroxyacetone. Nat. Commun..

[41] Vo T.-G., Kao C.-C., Kuo J.-L., Chiu C.-C., Chiang C.-Y. (2020). Unveiling the crystallographic facet dependence of the photoelectrochemical glycerol oxidation on bismuth vanadate. Appl. Catal. B Environ..

[42] Seadira T.W.P., Sadanandam G., Ntho T., Masuku C.M., Scurrell M.S. (2018). Preparation and characterization of metals supported on nanostructured TiO_2_ hollow spheres for production of hydrogen via photocatalytic reforming of glycerol. Appl. Catal. B Environ..

[43] Reddy N.L., Cheralathan K.K., Kumari V.D., Neppolian B., Venkatakrishnan S.M. (2018). Photocatalytic Reforming of Biomass Derived Crude Glycerol in Water: A Sustainable Approach for Improved Hydrogen Generation Using Ni(OH)_2_ Decorated TiO_2_ Nanotubes under Solar Light Irradiation. ACS Sustain. Chem. Eng..

[44] Lee Y., Kim S., Jeong S.Y., Seo S., Kim C., Yoon H., Jang H.W., Lee S. (2021). Surface-Modified Co-doped ZnO Photoanode for Photoelectrochemical Oxidation of Glycerol. Catal. Today.

[45] Kim S., An E., Oh I., Hwang J.B., Seo S., Jung Y., Park J.-C., Choi H., Choi C.H., Lee S. (2022). CeO_2_ nanoarray decorated Ce-doped ZnO nanowire photoanode for efficient hydrogen production with glycerol as a sacrificial agent. Catal. Sci. Technol..

[46] Yu J., Dappozze F., Martín-Gomez J., Hidalgo-Carrillo J., Marinas A., Vernoux P., Caravaca A., Guillard C. (2021). Glyceraldehyde production by photocatalytic oxidation of glycerol on WO_3_-based materials. Appl. Catal. B Environ..

[47] Yang L., Jiang Y., Zhu Z., Hou Z. (2022). Selective oxidation of glycerol over different shaped WO_3_ supported Pt NPs. Mol. Catal..

[48] Li Y., Mei Q., Liu Z., Hu X., Zhou Z., Huang J., Bai B., Liu L., Ding F., Wang Q. (2022). Fluorine-doped iron oxyhydroxide cocatalyst: Promotion on the WO_3_ photoanode conducted photoelectrochemical water splitting. Appl. Catal. B Environ..

[49] Monllor-Satoca D., Borja L., Rodes A., Gómez R., Salvador P. (2006). Photoelectrochemical behavior of nanostructured WO_3_ thin-film electrodes: The oxidation of formic acid. Chemphyschem.

[50] Iwai T. (1960). Temperature Dependence of the Optical Absorption Edge of Tungsten Trioxide Single Crystal. Phys. Soc. Jpn..

[51] Berak J.M., Sienko M. (1970). Effect of oxygen-deficiency on electrical transport properties of tungsten trioxide crystals. J. Solid. State Chem..

[52] Zhang J., Zhu G., Liu W., Xi Y., Golosov D.A., Zavadski S.M., Melnikov S.N. (2020). 3D core-shell WO_3_@ α-Fe_2_O_3_ photoanode modified by ultrathin FeOOH layer for enhanced photoelectrochemical performances. J. Alloys Compd..

[53] Dong P., Hou G., Xi X., Shao R., Dong F. (2017). WO_3_-based photocatalysts: Morphology control, activity enhancement and multifunctional applications. Environ. Sci. Nano.

[54] Nagy D., Szilágyi I.M., Fan X. (2016). Effect of the morphology and phases of WO_3_ nanocrystals on their photocatalytic efficiency. RSC Adv..

[55] Gillet M., Aguir K., Lemire C., Gillet E., Schierbaum K. (2004). The structure and electrical conductivity of vacuum-annealed WO_3_ thin films. Thin Solid Film..

[56] Miseki Y., Kusama H., Sugihara H., Sayama K. (2010). Cs-modified WO_3_ photocatalyst showing efficient solar energy conversion for O_2_ production and Fe (III) ion reduction under visible light. J. Phys. Chem. Lett..

[57] Fu J., Xu Q., Low J., Jiang C., Yu J. (2019). Ultrathin 2D/2D WO_3_/g-C_3_N_4_ step-scheme H_2_-production photocatalyst. Appl. Catal. B Environ..

[58] Hong S.J., Lee S., Jang J.S., Lee J.S. (2011). Heterojunction BiVO_4_/WO_3_ electrodes for enhanced photoactivity of water oxidation. Energy Environ. Sci..

[59] Hwang D.W., Kim J., Park T.J., Lee J.S. (2002). Mg-doped WO_3_ as a novel photocatalyst for visible light-induced water splitting. Catal. Lett..

[60] Cole B., Marsen B., Miller E., Yan Y., To B., Jones K., Al-Jassim M. (2008). Evaluation of Nitrogen Doping of Tungsten Oxide for Photoelectrochemical Water Splitting. J. Phys. Chem. C.

[61] Seabold J.A., Choi K.S. (2011). Effect of a cobalt-based oxygen evolution catalyst on the stability and the selectivity of photo-oxidation reactions of a WO_3_ photoanode. Abstr. Pap. Am. Chem. Soc..

[62] Cai L., Zhao J., Li H., Park J., Cho I.S., Han H.S., Zheng X. (2016). One-Step Hydrothermal Deposition of Ni:FeOOH onto Photoanodes for Enhanced Water Oxidation. ACS Energy Lett..

[63] Suen N.-T., Hung S.-F., Quan Q., Zhang N., Xu Y.-J., Chen H.M. (2017). Electrocatalysis for the oxygen evolution reaction: Recent development and future perspectives. Chem. Soc. Rev..

[64] McCrory C.C.L., Jung S., Peters J.C., Jaramillo T.F. (2013). Benchmarking heterogeneous electrocatalysts for the oxygen evolution reaction. J. Am. Chem. Soc..

[65] Deng H., Grunder S., Cordova K.E., Valente C., Furukawa H., Hmadeh M., Gándara F., Whalley A.C., Liu Z., Asahina S. (2012). Large-pore apertures in a series of metal-organic frameworks. Science.

[66] Furukawa H., Cordova K.E., O’Keeffe M., Yaghi O.M. (2013). The chemistry and applications of metal-organic frameworks. Science.

[67] Kitagawa S. (2014). Metal–Organic Frameworks (MOFs). Chem. Soc. Rev..

[68] Liao P.Q., Shen J.Q., Zhang J.P. (2018). Metal–organic frameworks for electrocatalysis. Coord. Chem. Rev..

[69] Greathouse J.A., Allendorf M.D. (2006). The interaction of water with MOF-5 simulated by molecular dynamics. J. Am. Chem. Soc..

[70] Kaye S.S., Dailly A., Yaghi O.M., Long J.R. (2007). Impact of preparation and handling on the hydrogen storage properties of Zn_4_O(1,4-benzenedicarboxylate)_3_ (MOF-5). J. Am. Chem. Soc..

[71] Low J.J., Benin A.I., Jakubczak P., Abrahamian J.F., Faheem S.A., Willis R.R. (2009). Virtual high throughput screening confirmed experimentally: Porous coordination polymer hydration. J. Am. Chem. Soc..

[72] Canivet J., Fateeva A., Guo Y., Coasne B., Farrusseng D. (2014). Water adsorption in MOFs: Fundamentals and applications. Chem. Soc. Rev..

[73] Nemiwal M., Gosu V., Zhang T.C., Kumar D. (2021). Metal organic frameworks as electrocatalysts: Hydrogen evolution reactions and overall water splitting. Int. J. Hydrogen Energy.

[74] Luo H., Zeng Z., Zeng G., Zhang C., Xiao R., Huang D., Lai C., Cheng M., Wang W., Xiong W. (2020). Recent progress on metal-organic frameworks based-and derived-photocatalysts for water splitting. Chem. Eng. J..

[75] Yu X.-Y., Feng Y., Guan B., Lou X.W.D., Paik U. (2016). Carbon coated porous nickel phosphides nanoplates for highly efficient oxygen evolution reaction. Energy Environ. Sci..

[76] Yang Y., Lun Z., Xia G., Zheng F., He M., Chen Q. (2015). Non-precious alloy encapsulated in nitrogen-doped graphene layers derived from MOFs as an active and durable hydrogen evolution reaction catalyst. Energy Environ. Sci..

[77] Kim S., Pena T.A.D., Seo S., Choi H., Park J., Lee J.H., Woo J., Choi C.H., Lee S. (2021). Co-catalytic effects of Bi-based metal-organic framework on BiVO_4_ photoanodes for photoelectrochemical water oxidation. Appl. Surf. Sci..

[78] Wang G., Sun Q., Liu Y., Huang B., Dai Y., Zhang X., Qin X. (2015). A bismuth-based metal-organic framework as an efficient visible-light-driven photocatalyst. Chemistry.

[79] Liu L., Zhang L., Wang F., Qi K., Zhang H., Cui X., Zheng W. (2019). Bi-metal-organic frameworks type II heterostructures for enhanced photocatalytic styrene oxidation. Nanoscale.

[80] Parthibavarman M., Karthik M., Prabhakaran S. (2018). Facile and one step synthesis of WO_3_ nanorods and nanosheets as an efficient photocatalyst and humidity sensing material. Vacuum.

[81] Katrib A., Hemming F., Wehrer P., Hilaire L., Maire G. (1995). The multi-surface structure and catalytic properties of partially reduced WO_3_, WO_2_ and WC + O_2_ or W + O_2_ as characterized by XPS. J. Electron. Spectrosc. Relat. Phenom..

[82] Nguyen V.H., Nguyen T.D., Van Nguyen T. (2020). Microwave-Assisted Solvothermal Synthesis and Photocatalytic Activity of Bismuth(III) Based Metal–Organic Framework. Top. Catal..

[83] Huang L.-W., Vo T.-G., Chiang C.-Y. (2019). Converting glycerol aqueous solution to hydrogen energy and dihydroxyacetone by the BiVO_4_ photoelectrochemical cell. Electrochim. Acta.

[84] Ahmed M.S., Jeon S. (2014). Synthesis and electrocatalytic activity evaluation of nanoflower shaped Ni-Pd on alcohol oxidation reaction. J. Electrochem. Soc..

[85] Du W., Mackenzie K.E., Milano D.F., Deskins N.A., Su D., Teng X. (2012). Palladium–tin alloyed catalysts for the ethanol oxidation reaction in an alkaline medium. ACS Catal..

[86] Houache M.S.E., Hughes K., Baranova E.A. (2019). Study on catalyst selection for electrochemical valorization of glycerol. Sustain. Energy Fuels.

[87] Liu Y., Wang M., Zhang B., Yan D., Xiang X. (2022). Mediating the Oxidizing Capability of Surface-Bound Hydroxyl Radicals Produced by Photoelectrochemical Water Oxidation to Convert Glycerol into Dihydroxyacetone. Acs Catal..

[88] Luo L., Chen W., Xu S.M., Yang J., Li M., Zhou H., Xu M., Shao M., Kong X., Li Z. (2022). Selective Photoelectrocatalytic Glycerol Oxidation to Dihydroxyacetone via Enhanced Middle Hydroxyl Adsorption over a Bi_2_O_3_-Incorporated Catalyst. J. Am. Chem. Soc..

[89] Çetinkaya S., Khamidov G., Özcan L., Palmisano L., Yurdakal S. (2022). Selective photoelectrocatalytic oxidation of glycerol by nanotube, nanobelt and nanosponge structured TiO_2_ on Ti plates. J. Environ. Chem. Eng..

[90] de Escobar C.C., Lansarin M.A., Santos J.H.Z.D., Brandestini M.D. (2018). Molecularly imprinted photocatalyst for glyceraldehyde production. J. Sol-Gel Sci. Technol..

[91] Ouyang J., Liu X., Wang B.H., Pan J.B., Shen S., Chen L., Au C.T., Yin S.F. (2022). WO_3_ Photoanode with Predominant Exposure of {202} Facets for Enhanced Selective Oxidation of Glycerol to Glyceraldehyde. ACS Appl. Mater. Interfaces.

