# Supplementary material for: Photoelectrochemical Selective Oxidation of Glycerol to Glyceraldehyde with Bi-Based Metal–Organic-Framework-Decorated WO3 Photoanode
Source: Nanomaterials (Basel). 2023 May 21;13(10):1690. doi: 10.3390/nano13101690 (PMC10223667; doi:10.3390/nano13101690)
Supplement: Supplementary file 1 [file nanomaterials-13-01690-s001.zip › nanomaterials-2415421-supplementary.pdf]

Supporting Information

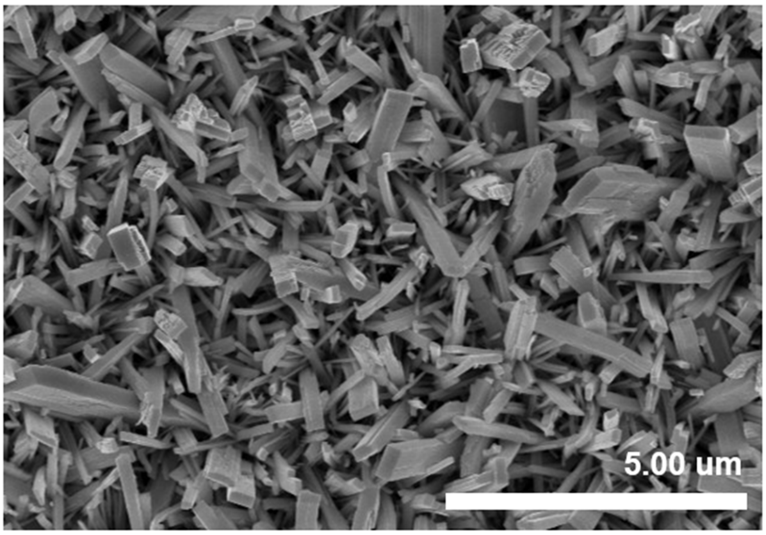

Figure S1. Top-view SEM images of WO_3 on FTO.

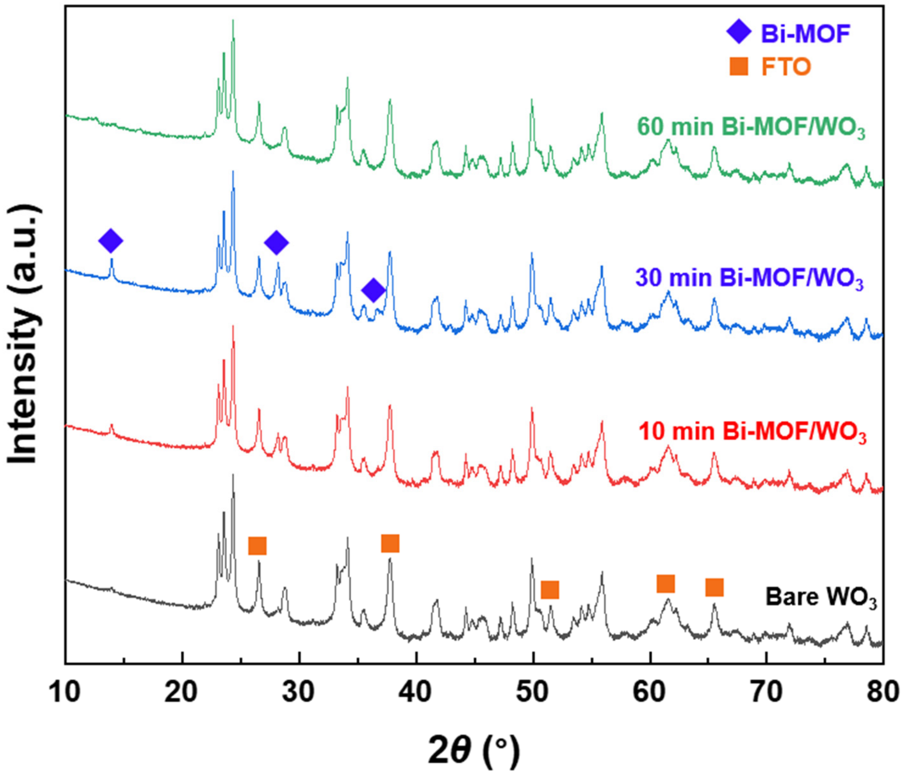

Figure S2. Out-of-plane θ–2θ scans of Bi-MOF/WO_3 at different synthesis times of MOF.

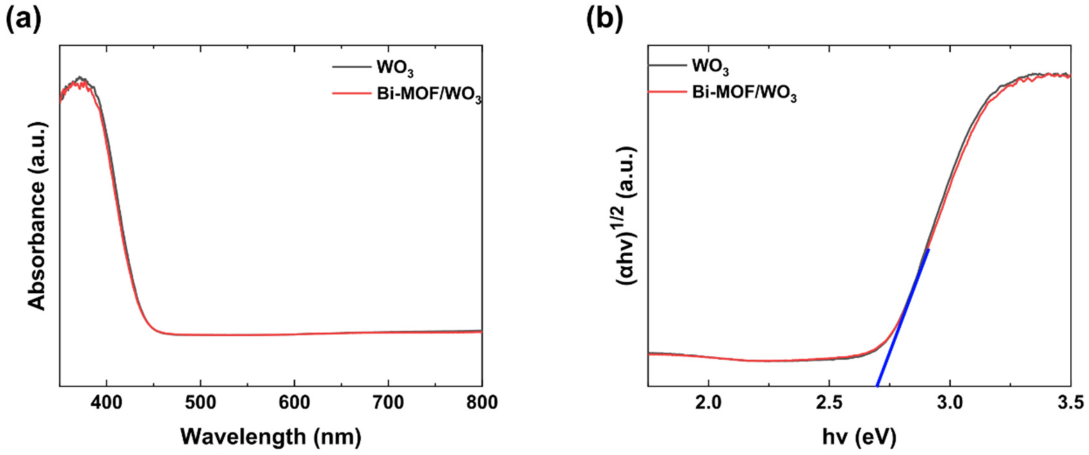

Figure S3. (a) UV-vis absorbance spectra, (b) Tauc plot

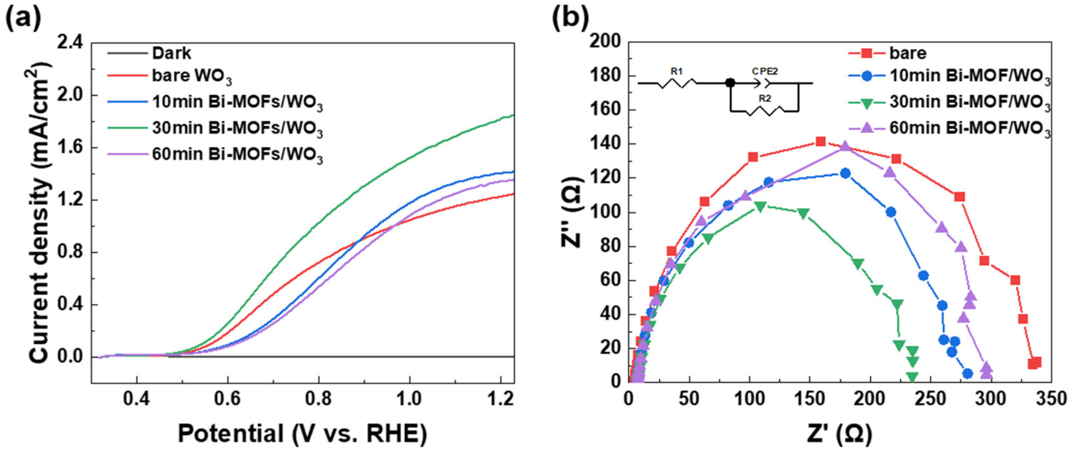

Figure S4. PEC performance of WO_3 and Bi-MOF/WO_3 in 0.5 M Na_2SO_4 electrolytes, with pH adjusted to 2 using sulfuric acid, with 0.1 M glycerol. (a) LSV polarization curves, (b) Nyquist plots with different synthesis times of MOF.

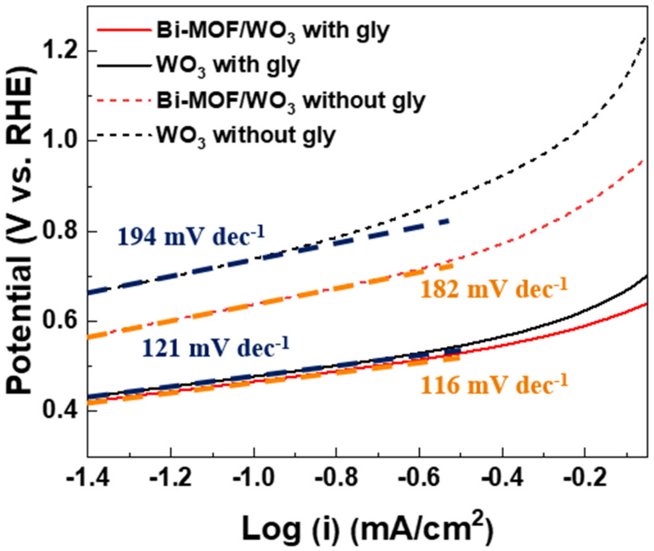

Figure S5. Tafel plots of WO_3 and Bi-MOF/WO_3 in 0.5 M Na_2SO_4 electrolytes, with pH adjusted to 2 using sulfuric acid, with and without 2 M glycerol.

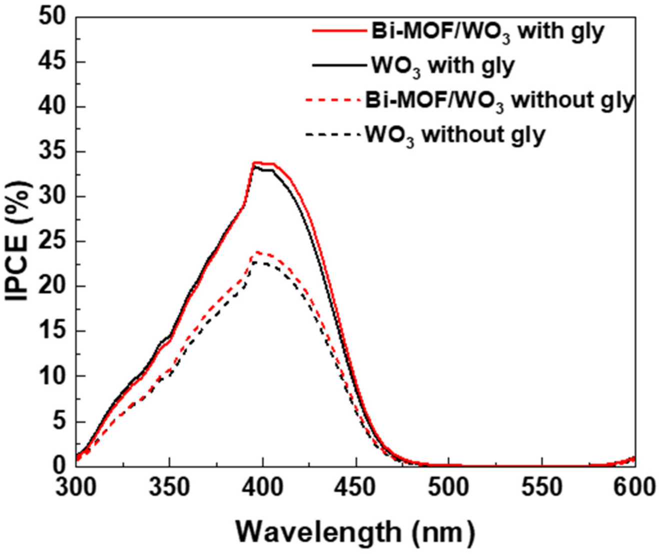

Figure S6. IPCE values at 1.2 V *vs*. RHE with 2 M glycerol.

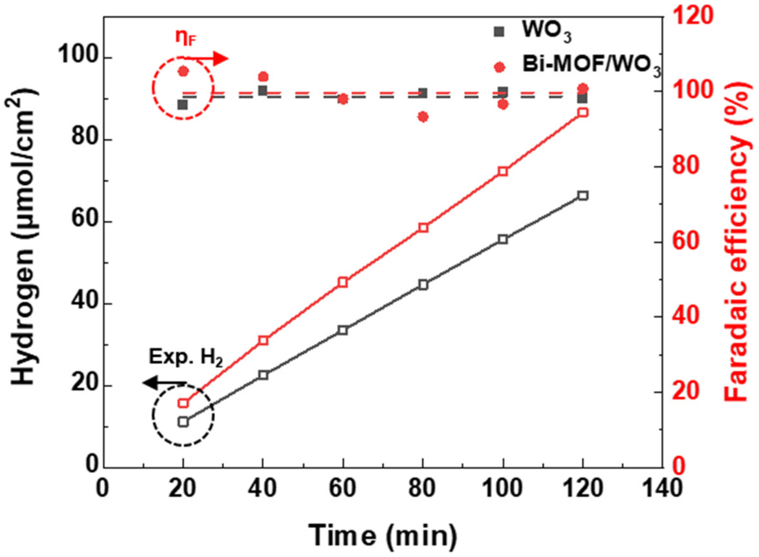

Figure S7. Faradaic efficiency and production rate of hydrogen at 1.2 V *vs*. RHE

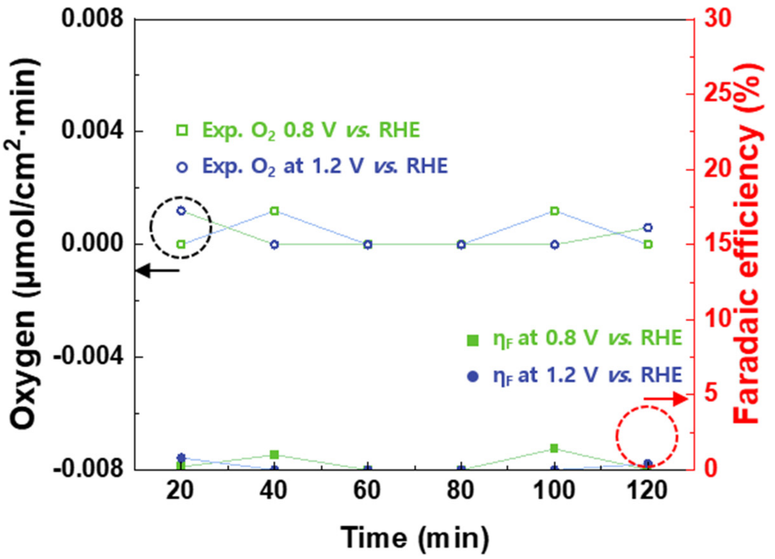

Figure S8. Faradaic efficiency and production rate of oxygen.

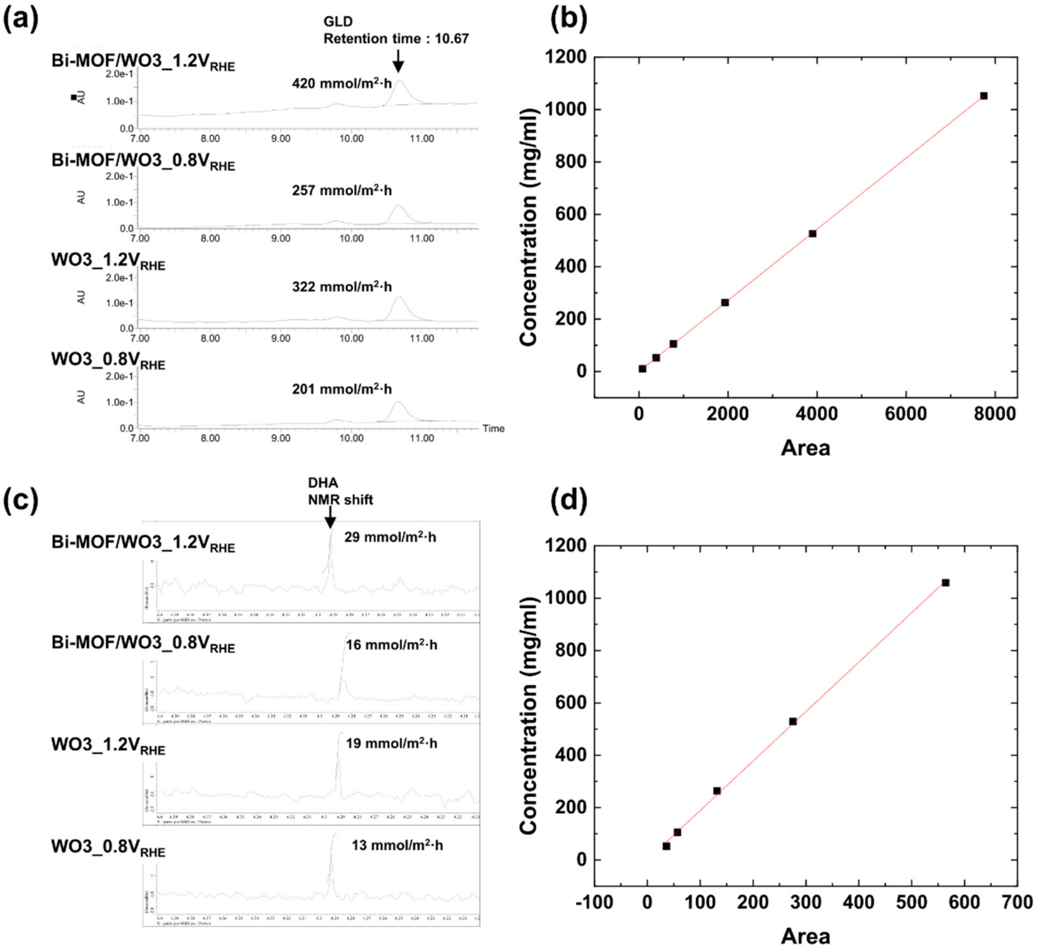

Figure S9. (a) Liquid chromatography of produced glyceraldehyde, (b) calibration curve of standard glyceraldehyde (c) NMR spectra of produced dihydroxyacetone, (d) calibration curve of standard dihydroxy acetone.

Table S1. Reaction rate, production rate, and faradaic efficiency from WO_3 and Bi-MOF/WO_3.

Electrode	Applied potential (V_{RHE})	Glycerol	Glyceraldehyde		Dihydroxyacetone	
		Conversion rate (mmol/m^2, µmol/cm^2)	Production rate (mmol/m^2, µmol/cm^2)	Faradaic efficiency (%)	Production rate (mmol/m^2, µmol/cm^2)	Faradaic efficiency (%)
WO_3	0.8	215<br>21.5	201<br>20.1	94	13<br>1.3	6
WO_3	1.2	356<br>35.6	322<br>32.2	90	19<br>1.9	5
Bi-MOF/WO_3	0.8	278<br>27.8	257<br>25.7	93	17<br>1.7	6
Bi-MOF/WO_3	1.2	467<br>46.7	420<br>42.0	90	29<br>2.9	6

Table S2. Photocatalyst, and photoelectrode for the selective oxidation of glycerol.

Material	Medium (total volume)	Time	Major product	FE (%)	Selectivity (%)	Ref.
$BiVO_4$	0.1 M glycerol, 0.5 M Na_2SO_4	1 h	DHA	50	50	[40]
$BiVO_4$	0.1 M glycerol, 0.1 M NaBi	2 h	DHA	-	60	[41]
Ag/LDH/ TiO_2	0.1 M glycerol, 0.5 M Na_2SO_4	4 h	DHA	55	72	[87]
Bi_2O_3 /TiO_2	0.1 M glycerol, 0.5 M Na_2SO_4, H_2SO_4	1h	DHA	62	75	[88]
TiO_2	10 mM glycerol, 20 mM Na_2SO_4	4 h	GAD	-	44	[89]
H-WO_3 /TiO_2	Na_2SO_4, NaBi,	1 h	GAD	51	56	[90]
WO_3	1 M glycerol, 0.1 M Na_2SO_4	2 h	GAD	75	80	[91]
Bi-MOF/ WO_3	2 M glycerol, 0.5 M Na_2SO_4,	1 h	GAD	93	94	This work
